# Interaction landscape of a ‘C^α^NN’ motif with arsenate and arsenite: a potential peptide-based scavenger of arsenic†

**DOI:** 10.1039/c8ra08225a

**Published:** 2019-01-09

**Authors:** Subhankar Sahu, Tridip Sheet, Raja Banerjee

**Affiliations:** Department of Biotechnology and Head Department of Bioinformatics, Maulana Abul Kalam Azad University of Technology, West Bengal (Formerly Known as West Bengal University of Technology) BF-142, Salt Lake Kolkata 700064 West Bengal India ban_raja@yahoo.com banraja10@gmail.com

## Abstract

Arsenic (As) is a toxic metalloid that has drawn immense attention from the scientific community recently due to its fatal effects through its unwanted occurrence in ground water around the globe. The presence of an excess amount of water soluble arsenate and/or arsenite salt (permissible limit 10 μg L^−1^ as recommended by the WHO) in water has been correlated with several human diseases. Although arsenate (HAsO_4_^2−^) is a molecular analogue of phosphate (HPO_4_^2−^), phosphate is indispensable for life, while arsenic and its salts are toxic. Therefore, it is worthwhile to focus on the removal of arsenic from water. Towards this end, the design of peptide-based scaffolds for the recognition of arsenate and arsenite would add a new dimension. Utilizing the stereochemical similarity between arsenate (HAsO_4_^2−^) and phosphate (HPO_4_^2−^), we successfully investigated the recognition of arsenate and arsenite with a naturally occurring novel phosphate binding ‘C^α^NN’ motif and its related designed analogues. Using computational as well as biophysical approaches, for the first time, we report here that a designed peptide-based scaffold based on the ‘C^α^NN’ motif can recognize anions of arsenic in a thermodynamically favorable manner in a context-free system. This peptide-based arsenic binding agent has the potential for future development as a scavenger of arsenic anions to obtain arsenic free water.

## Abstract 2

Potential peptide-based scavenger of toxic metalloid arsenic causing fatal effects through its unwanted occurrence in ground water around the globe.

## Graphical abstract

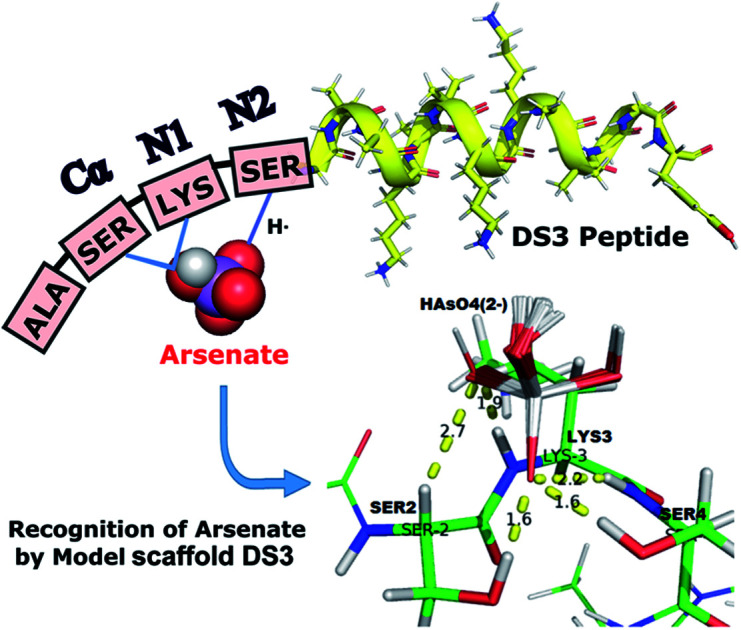

## 1 Introduction

Arsenic (As) contamination in ground water is a global health issue.^1–5^ It is one of the largest worldwide health disasters affecting around 150 million people who are at high risk.^6^ As per reported investigations, India and Bangladesh are the most victimized countries.^7–11^ Arsenic having similar chemical properties with phosphorus, is a pnictogen element and a member of group 15 of the modern periodic table.^12^ The most favorable oxidation states of As are +5 and +3. AsO_4_^3−^, HAsO_4_^2−^ and H_2_AsO_4_^1−^ are formed from arsenic acid (H_3_AsO_4_; oxidation state of As +5), while AsO_3_^3−^, HAsO_3_^2−^ and H_2_AsO_3_^1−^ are formed from arsenious acid (H_3_AsO_3_; oxidation state of As +3). From a chemistry point of view, it can be inferred that arsenate (HAsO_4_^2−^) should behave as a molecular analogue of phosphate (HPO_4_^2−^) and indeed, it is found that both compete for different biological processes.^13^ Although both phosphorus and arsenic carry almost the same electronegativity, the atomic radius of arsenic is larger.^12^ Unlike phosphorous, compounds of arsenic are highly toxic. However, the mechanisms of arsenic toxicity in living organisms have not been extensively deciphered. Phosphate (HPO_4_^2−^) is indispensable for life, as it regulates several biological functions^14,15^ and is present as an important constituent of essential biological macromolecules (*e.g.*, DNA, lipids, *etc.*). On the contrary, arsenic is toxic and causes hindrance in operations of processes like inhibition of ATP synthesis during oxidative phosphorylation and many others.^13,16–19^ Most arsenic pollution goes right through ground water as excess water soluble arsenate and/or arsenite salts (permissible limit 10 μg L^−1^ as recommended by WHO). Pentavalent and trivalent states of arsenic, along with their methylated derivatives, interfere with the metabolic pathway and normal cellular activity, but the exact mechanism is still obscure. Recently, arsenic contamination in water has been found to be linked with numerous human diseases^20,21^ which include cardio vascular abnormalities,^22–25^ risk of malignancy,^3,26–28^ diabetes,^29–31^ gastrointestinal anomalies,^4,5^ nervous system dysfunction,^32–34^ respiratory distress and obstructive lung diseases,^35,36^ dermatological lesions,^37,38^*etc.* At this point, it is worthy to focus on aspects of arsenic removal from water. However, it should be noted that removal of arsenite (As^+3^) from water is more difficult than arsenate (As^+5^).^39^ Available purification techniques range from co-precipitation with naturally occurring iron^40,41^ to a three pitcher (3-kolshi) filtration unit.^42^ Recombinant DNA technologies also have been employed to develop an arsenic-binding DNA aptamer for arsenic removal.^43^ Although various approaches have been adopted globally for removing As derivatives from water, molecular recognition of As anions by proteins and peptides at the atomic scale is a new approach worth exploring.

Attempts to remove arsenic-based anions utilizing their recognition by peptide-based scaffolds would add a new dimension. However, identification of a proper peptide motif which can recognize anions of As in a context-free situation is the real challenge. Among several anion binding peptide motifs, the ‘C^α^NN’ motif,^44^ responsible for binding phosphate in protein, would be a logical choice for recognizing arsenic salts (arsenate/arsenite), because the arsenate ion is stereochemically similar to phosphate and sulfate. The ‘C^α^NN’ motif, found mostly in the N-terminal of helices,^44^ is a three residue motif capable of recognizing an anion (mainly phosphate and sulfate) even in a context-free non-proteinaceous environment.^45–47^ Cα_*i*−1_–H, N_*i*_–H and N_*i*+1_–H of the backbone atoms in the three consecutive residues of the motif sequence participate in anion binding as mediated through hydrogen bonding (H-bond). It is established that Cα_*i*−1_ participates in a relatively weak hydrogen bond, while N_*i*_ and N_*i*+1_ form rather a strong one. Further, the ‘C^α^NN’ motif bears a ‘GXX’ sequence pattern with a conserved geometry of βαα or βαβ and shows higher affinity towards sulfate (SO_4_^2−^) ions than phosphate (HPO_4_^2−^) ions in a context-free system.^46,47^ Their ability to recognize anions has been characterized extensively through *in silico* approaches like molecular docking^46^ and molecular dynamics simulation^46,48^ along with quantum mechanical calculations^48^ and has been further validated *via* complementary biophysical techniques like NMR and Circular Dichroism Spectroscopy.^45,47^ All these experiments establish that efficiency of anion binding largely depends on the ‘C^α^NN’ involving residues and nature of the anion.

In this study, we explored potential peptide-based scaffolds that would recognize arsenate and arsenite compounds. Intrinsic affinity of the ‘C^α^NN’ motif for HPO_4_^2−^ ion and the stereochemical similarity between HPO_4_^2−^ and HAsO_4_^2−^ are the principal guiding factors of our present investigation. We used a group of arsenic compounds as anionic ligands to scrutinize the potentiality of anion recognition ability with pre-designed model scaffolds based on the ‘C^α^NN’ anion binding motif by analyzing the interaction landscape. Our results clearly indicate that the designed peptide-based scaffolds can recognize the anions of As in a thermodynamically favorable manner in a context-free system and thus would be a potential candidate to be used as a scavenger of arsenate/arsenite in a suitable form if attached to a water filtration unit for As removal.

## 2 Methods

### 2.1 Library of ligands used

In aqueous solution arsenic forms oxo-anions like arsenite (salts of H_3_AsO_3_ acid) and arsenate (salts of H_3_AsO_4_ acid) with oxidation states +3 and +5, respectively.^12^ Respective p*K*_a_ values of arsenous acid (H_3_AsO_3_) are 9.23 (p*K*_a1_), 12.13 (p*K*_a2_), and 13.40 (p*K*_a3_), while for arsenic acid (H_3_AsO_4_) they are 2.20 (p*K*_a1_), 6.97 (p*K*_a2_), and 11.53 (p*K*_a3_)^12^ which are comparable to the p*K*_a_ values of phosphoric acid (H_3_PO_4_). A set of arsenic compounds were used for the molecular docking study as ligands (anions) which included AsO_4_^3−^, HAsO_4_^2−^ and H_2_AsO_4_^1−^ from the arsenic acid group and H_2_AsO_3_^1−^, HAsO_3_^2−^ and AsO_3_^3−^ from the arsenous acid group. Three dimensional coordinates of arsenate (HAsO_4_^2−^) and arsenite (AsO_3_^3−^) were retrieved from RCSB PDB^49^ with their three letter codes, “ART” (https://www3.rcsb.org/ligand/ART)^50^ and “AST” (https://www3.rcsb.org/ligand/AST),^51^ respectively. Other related derivatives of As compounds were generated with the help of GaussView 5 molecular builder and visualizer^52^ by adding or removing required hydrogen atom(s), providing suitable net charge, and finally optimizing at the semi-empirical PM6 level.^53^ Sulfate and phosphate ions additionally were used to compare the effectiveness of binding arsenic derivatives. Because arsenic anions interfere with the metabolic pathway and normal cellular activity, priority was given to those conjugate bases of arsenic acids which exist at the physiological pH range (pH 6–8) *i.e.*, arsenate (as HAsO_4_^2−^, p*K*_a2_: 6.97) and thus results related to arsenite (with p*K*_1_: 9.23) are beyond the scope of this discussion.

### 2.2 Sequences of model peptides

Conservation of sequence in the ‘C^α^NN’ motif^44^ was observed from analysis of different fold representative structures of proteins. A series of chimeric peptides were constructed, where the naturally occurring ‘C^α^NN’^44^ or designed model scaffolds based on the ‘C^α^NN’ peptide sequences^48^ were appended at the N-terminus of a model context-free helix (ABGY).^54,55^ All the scaffolds used here have extensive investigation portfolios with both sulfate and phosphate ions as interacting partners.^46–48^ In the present study, arsenic related ligands were used to extract information about anion recognition by these scaffolds to explore whether they would act as effective scavengers for anion(s) of arsenic (As). Details of the peptides used are listed here where the underline segment represents the anion binding sequences having either the naturally occurring ‘C^α^NN’^44^ or designed model scaffolds based on the ‘C^α^NN’ peptide sequences^48^ (constituent amino acids are designated through three letter symbols).

DS1: Ac-Ala-**Gly-Lys-Ser**-Ala-Aib-Ala-Lys-Ala-Aib-Lys-Ala-Lys-Ala-Aib-Gly-Gly-Tyr-NH2.

DS3: Ac-Ala-**Ser-Lys-Ser**-Ala-Aib-Ala-Lys-Ala-Aib-Lys-Ala-Lys-Ala-Aib-Gly-Gly-Tyr-NH2.

CPS224Ac: Ac-Leu-**Gly-Lys-Gln**-Ala-Aib-Ala-Lys-Ala-Aib-Lys-Ala-Lys-Ala-Aib-Gly-Gly-Tyr-NH2.

CPS226: Ac-Gly-**Ser-Ala-Lys**-Ala-Aib-Ala-Lys-Ala-Aib-Lys-Ala-Lys-Ala-Aib-Gly-Gly-Tyr-NH2.

SCPS224Ac: Ac-Leu-**Gly-Lys-Gln**-Ala-NH2.

SCPS226: Ac-Gly-**Ser-Ala-Lys**-Ala-NH2.

The N-terminals of all the peptides are capped with an acetyl (Ac) group while the amide (–CONH) group is used to protect the respective C-terminus. For all the following larger peptides sequences, the motif (after 4^th^ residue) is identical and essentially fixed to a right handed helical peptide (ABGY).^54,55^ α-Aminoisobutyric acid (Aib) was included to increase helix stability because of its high helix inducing propensity. The ‘C^α^NN’ motif sequences of CPS224Ac, SCPS224Ac, and CPS226, SCPS226 were obtained from the crystal structures 1MUG^14^ and 1YCC,^56^ respectively from PDB. Peptide capping was done *via* Discovery Studio Visualizer 4.1.^57^ CPS224Ac and CPS226 were demonstrated as natural ‘C^α^NN’ motif-containing peptides whereas DS1 and DS3 represent designed scaffolds.^48^

### 2.3 Molecular docking

Bio-molecular interactions among the peptides and ligands were primarily investigated and analyzed *via* the AutoDock 4.2 molecular docking package.^58–61^ MGL Tools 1.5.6 (ref. 62) was installed in Windows 7 OS and used for preparing different parameter files for docking purposes. Arsenic as a molecule is non-recognizable to the AutoDock program, so, parameterization was carried out by the standard procedure mentioned in the AutoDock official webpage (http://autodock.scripps.edu/faqs-help/how-to/adding-new-atom-parameters-to-autodock). Further manual additions of the required parameters (*viz* van der Waals radius, atomic solvation volume, *etc.*) were accomplished through ‘AD4_parameters.dat’ and ‘AD4.1_bound.dat’ files while keeping those files in the working directory where we ran the docking program. Parameters used for the arsenic atom are Rii (sum of van der Waals radius of two like atoms, in Å): 4.30, epsii (vdw well depth in kacl mol^−1^): 0.200, vol (atomic solvation volume, Å^3^): 41.6087, and solpar (atomic solvation parameter): −0.00110. Addition of the lines ‘parameter_file AD4_parameters.dat’, and ‘parameter_file AD4.1_bound.dat’ without quotes (which instructs AutoDock to read the parameter files from a default location) was done at the beginning of the .gpf (grid parameter file) and .dpf (docking parameter file) before running *autogrid4* and *autodock4* programs, respectively. All the coordinates of ligands and peptide were loaded into AutoDock Tools 1.5.6 (available under MGL Tools) to add polar hydrogens. Partial charge was calculated and saved in ‘.pdbqt’ format. For all the arsenic compounds, partial charge was calculated using the Gaussian 2009 software package^63^*via* calculating the ground state energy in semi-empirical mode (as AutoDock also uses a semi empirical free energy force field) with a PM6 Hamiltonian basis set^64^ and saved in a ‘.pdbqt’ format. Distribution of partial charge used for docking purposes with HAsO_3_^2−^ and HAsO_4_^2−^ is mentioned in ESI Fig. S1.† Size of the gird box was set [with grid points 44(*x*) 44(*y*) 44(*z*)] in such a way that it included up to the Lys8 residue from the N terminal Ac-group. After generating the map files for rigid docking from the grid parameter file, final docking was carried out by a genetic algorithm approach.^59^ The maximum number of energy evaluations was set to 2.5 × 10^7^ with a rate of gene mutation 0.02 and rate of crossover 0.8 for the required search parameters of the genetic algorithm and 250 conformations (number of GA runs) were generated to find all the ligand binding states. Results were validated through AutoDock Tools and also by manually written scripts by extracting information from the docking log files. Education PyMOL (version 1.3)^65^ was used to produce the images.

### 2.4 Mass spectroscopy

Molecular association of arsenate with peptide DS3 was experimentally determined by investigating the molecular mass (*m*/*z*) of the free-peptide and arsenate-bound peptide separately. Using MALDI-MS (Autoflex III TOF/TOF 200), the molecular mass of peptide DS3 (MW of DS3 1704) was obtained in the positive mode in H_2_O/CH_3_CN (1 : 1) with 0.1% HCOOH. On the other hand, the molecular mass of the arsenate added (added as Na_2_HAsO_4_) species of DS3 was determined from the corresponding *m*/*z* value in the negative mode of ESI-FTMS (Apex Ultra 70, Bruker Daltonics direct infusion mode), in H_2_O/CH_3_CN (1 : 1) with 0.1% NH_3_.

### 2.5 Circular dichroism spectroscopy

The conformational landscape of peptide DS3 upon interaction with arsenate in the fully aqueous condition was investigated using far-UV CD spectra (range of wavelength 250–190 nm). At room temperature (25 °C) before and after addition of arsenate (added as Na_2_HAsO_4_), the spectra were recorded using a Jasco J-815 CD-spectropolarimeter. Concentration of the peptide was kept at ∼75 μM (measured from Tyr absorbance at 275 nm, *ε* = 1450 cm^−1^ M^−1^) and arsenate was added at a ratio of 1 : 3 (peptide : arsenate). The CD spectra were recorded in a 1 mm path length cuvette and reported as mean residue ellipticity (deg cm^2^ dmol^−1^).

### 2.6 Isothermal titration calorimetric

In order to measure the binding affinity of arsenate for peptide DS3, along with the associated thermodynamic parameters of the interaction, isothermal titration calorimetric (ITC) data were recorded using a Microcal ITC200 (Malvern) at 25 °C. A 400 μL aqueous solution of peptide DS3 with a concentration of ∼50 μM was kept in the reaction cell and titrated by adding 1.5 mM arsenate solution (1 : 30 ratio of peptide and arsenate). As a control, a similar titration was carried out only with HPLC water (without the peptide) and that data was subtracted from the data obtained using the peptide.

### 2.7 Molecular dynamics simulation

To pursue a molecular dynamics simulation, we selected the ligand bound conformation generated from the molecular docking process of DS3 and HAsO_4_^2−^ (for reasons described in Discussion section). Simulation was done with the help of the GROMACS 5.1.2 package^66^ using Charmm27 force field^67,68^ within a cubic box in an explicitly water solvated system. Arsenate (HAsO_4_^2−^) was properly parameterized to keep its behavior consistent with the charmm27 force field. Ligand “AST” (HAsO_4_^2−^) was taken from PDB [https://www3.rcsb.org/ligand/AST] and, with the help of GaussView 5 (ref. 52) and Gaussian09 (ref. 63) programs, its geometry optimization along with partial charge distribution were calculated with the B3YLP level and the 6-31G++ basis set.^69^ Bonded and non-bonded parameters, adapted from the article “Structural and Functional Consequences of Phosphate–Arsenate Substitutions in Selected Nucleotides: DNA, RNA, and ATP,”^70^ was suitably added to the charmm27 force-field files within the installed GROMACS distribution. The simulation box had to be large enough, for which distance of the periphery of the peptide and the side of the box was kept approximately 2 nm. Pre-equilibration of the system was done with simple point charge (SPC) water model molecules.^71^ The system was neutralized by adding the requisite numbers of sodium or chloride ions and then its energy was minimized using a steepest descent algorithm. NVT (constant number of particles, volume, and temperature) and NPT (constant number of particles, pressure, and temperature)^72^ simulations were carried out (100 ps for both NVT and NPT) as part of the standard procedure for equilibration of the system. Finally, the equilibrated system was allowed to dynamically evolve up to 50 ns and the information from it was saved at every 10 ps interval to generate an ensemble of conformational states. Constraint was applied to all bonds with the LINCS algorithm^73^ and the non-bonded interaction was taken care of by PME method.^74^ Upon completion of the 50 ns simulation, time dependent behavior of ligand (HAsO_4_^2−^) with the designed scaffold (DS3) was analyzed by the default GROMACS utilities and custom written Perl scripts as a function of time.

## 3 Results and discussion

### 3.1 Molecular docking experiment

A molecular docking experiment enabled us to ensure thermodynamic feasibility of recognizing the anions comprising As (oxidation states +3 and +5) with the peptide scaffolds. The experimental output corroborated affinity as well as the potential of binding different ligands with the peptide-based scaffolds. Long 18 residue peptides (*e.g.*, DS1, DS3, CPS224Ac, and CPS226) along with the short sequences (*e.g.*, SCPS224Ac and SCPS226) containing only the ‘C^α^NN’ fragment were allowed separately to interact with each of the ligands from the library (as mentioned in Methods). Besides the binding free energy, a few other parameters (described in following sections) were also used to infer the ligand binding quality of the peptides.

### 3.2 Binding free energy based analysis

The respective binding free energy values obtained from the molecular docking experiment clearly emphasized that all of the peptides used have significant affinity towards the ligands of arsenic, as is similar to sulfate and phosphate ions. Appearance of a ‘single cluster’ of the interaction energy (binding free energy obtained from the MGL Tools [version 1.5.6]) for all the individual peptides with their respective ligands of As suggested that a single site of the peptide is responsible for recognizing the As anions. In all cases, interactions were observed between the arsenic ligands and the ‘C^α^NN’ segment of the peptides, where the interactions are mediated through typical hydrogen bonds between the Cα_*i*−1_–H; N_*i*_–H; N_*i*+1_–H and oxygen atoms of the respective As ligand similar to that of sulfate and the phosphate ions.^46,48^ It is quite appealing to note that in spite of the presence of charged side-chains with lysine residues that in all cases only the ‘C^α^NN’ segment interacts with the As-ligands as is also found for the sulfate and phosphate ions.^46,48^ However, potentiality of the interactions varies (Table 1) depending on the type of the ligand, its geometry, and charge as well as the sequences of the ‘C^α^NN’ segment of the peptides. The designed scaffolds [DS1: **Gly2-Lys3-Ser4** and DS3: **Ser2-Lys3-Ser4**] show comparatively higher anion recognition ability (Table 1) in terms of binding free energy for the ligands of As derivatives over the sequences containing the natural ‘C^α^NN’ motif sequence [CPS224Ac: **Gly2-Lys3-Gln4** and CSP226: **Ser2-Ala3-Lys4**], as similar to interactions observed for the SO_4_^2−^ and HPO_4_^2−^ ions^48^ (ESI Fig. S2†). Out of all the participating scaffolds, DS3 showed the most favourable interaction with all the anions, having a binding free energy of −3.08 kcal mol^−1^, −3.8 kcal mol^−1^, −3.13 kcal mol^−1^, and −2.92 kcal mol^−1^ for HAsO_4_^2−^, HAsO_3_^2−^, HPO_4_^2−^ and H_2_AsO_3_^−^ ions respectively (Table 1). This is at par with the observation obtained with the SO_4_^2−^ and HPO_4_^2−^ ions.^48^ Although both of these ligands (HAsO_3_^2−^ and HAsO_4_^2−^) are potent poisons and silent killers through pollution in groundwater, HAsO_3_^2−^ is often considered to be more lethal than arsenate (HAsO_4_^2−^). It is quite appealing to note that in terms of binding free energy, HAsO_3_^2−^ was better recognized by DS3 in comparison to HAsO_4_^2−^ (Table 1). One reason for a higher free energy of binding for arsenite over arsenate (having same net charge of −2) can be speculated as follows. In comparison to HAsO_4_^2−^, distributed partial charges with oxygen atoms are somewhat higher in HAsO_3_^2−^ due to the lower oxidation state of As (+3) thus enhancing the affinity of interaction (charge distribution of an arsenate and arsenite ligand can be seen in ESI Fig. S1†). Both As and P have almost comparable electronegativity, while the quickly noticeable difference is the atomic radius (As > P) due to the presence of an inner line d orbital in As. The overall observation leads one to conclude that the designed peptide scaffolds (DS1 and DS3) can be considered as potent candidates to bind HAsO_3_^2−^ and HAsO_4_^2−^, the interactions of which are comparable to that of HPO_4_^2−^.

**Table 1:** Comparison of free energy of binding of the respective ligands with the corresponding peptides expressed in kcal mol^−1^ indicates that DS3 shows higher affinity. Values of the inhibition constant obtained from the AutoDock program are listed in parentheses

	HPO_4_^2−^	HAsO_4_^2−^	HAsO_3_^2−^	H_2_AsO_3_^1−^
DS1	−2.51 (14.19 mM)	−2.51 (14.22 mM)	−3.09 (5.49 mM)	−2.58 (11.62 mM)
DS3	−3.13 (4.76 mM)	−3.08 (5.21 mM)	−3.8 (1.53 mM)	−2.92 (6.33 mM)
CPS224Ac	−1.66 (59.08 mM)	−1.71 (54.60 mM)	−2.37 (17.11 mM)	−1.67 (51.28 mM)
CPS226	−1.97 (33.81 mM)	−2.02 (32.09 mM)	−2.78 (8.81 mM)	−2.25 (39.77 mM)
SCPS224Ac	−2.39 (16.29 mM)	−2.47 (15.03 mM)	−3.43 (2.71 mM)	−2.49 (13.43 mM)
SCPS226	−1.72 (52.18 mM)	−1.77 (49.64 mM)	−2.54 (13.30 mM)	−2.14 (26.30 mM)

### 3.3 Quantitative analysis of peptide and ligand interaction

**Fig. 1:**
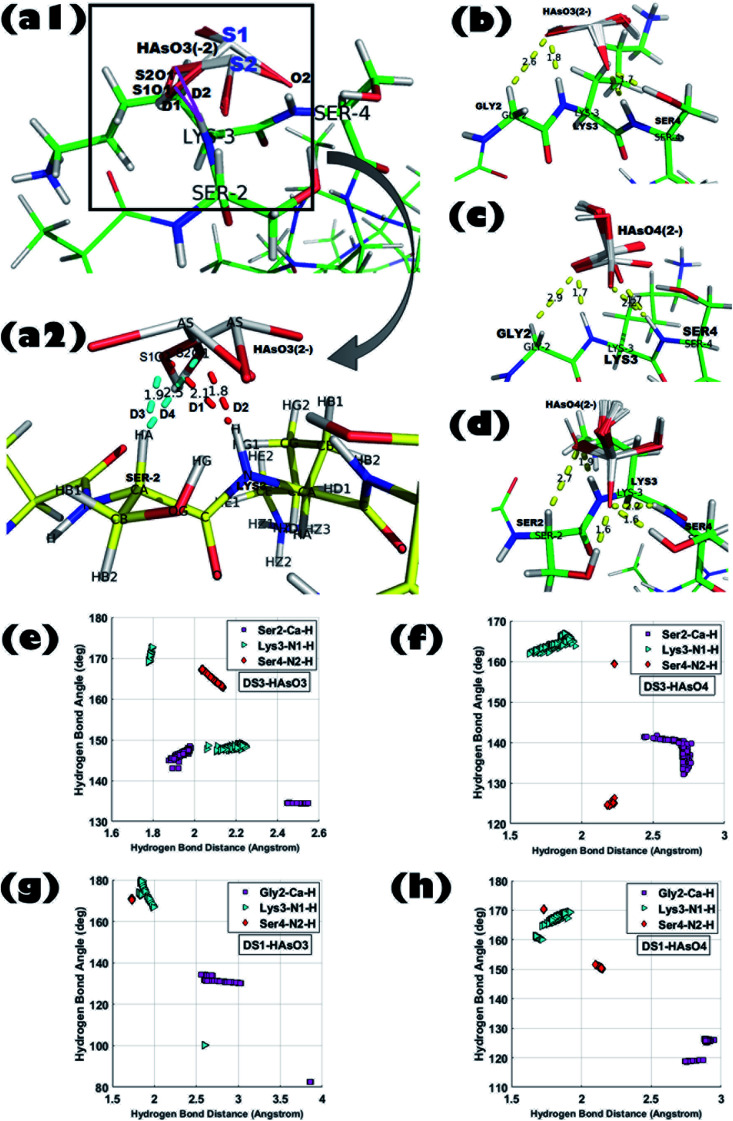
Superposition of all 250 ligand conformations generated from interactions between peptide scaffold and the respective anion obtained from Molecular Docking experiments; (a1) DS3 (Ser2-Lys3-Ser4) : HAsO_3_^2−^ [ligand binding conformations are shown in zoomed view by taking one ligand conformer from each state in figure (a2). Distances of the S1O1 and S2O1 atom from the Lys3-N–H and Ser2-Cα–H atom are shown in red and cyan colors, respectively]. (b) DS1 (Gly2-Lys3-Ser4) : HAsO_3_^2−^, (c) DS1 (Gly2-Lys3-Ser4) : HAsO_4_^2−^, and (d) DS3 (Ser2-Lys3-Ser4) : HAsO_4_^2−^; H-bond distance and angle distribution of ‘C^α^NN’ motif residues of DS3 peptide with the respective anions in (e), (f), ‘C^α^NN’ motif residues of DS1 peptide with the respective anions in (g) and (h).

The model host helix ABGY provides a native environment to the ‘C^α^NN’ motif which is appended at the N-terminus of ABGY. Interactions of anions of As with the peptide-scaffolds mediated through a hydrogen bond (H-bond) were analyzed by statistical distributions of the (X)–H⋯O bond length and X–H⋯O bond angle. Hydrogen bond distance between (Cα)H⋯O, (N1)H⋯O, and (N2)H⋯O atoms along with the angle formed by Cα–H⋯O, N1–H⋯O, and N2–H⋯O atoms were extensively analyzed for all the 250 poses generated for each individual interaction in order to portray the binding of peptide and ligand. Detailed interaction parameters of HAsO_4_^2−^ and HAsO_3_^2−^ with DS3 and DS1 are shown in Fig. 1. Distribution of distance and angles between atoms of the ‘C^α^NN’ segment with the individual ligands (HAsO_4_^2−^ and HAsO_3_^2−^) are shown *via* a histogram plot for which the average (*μ*) and standard deviation (*s*) are noted (ESI Fig. S3 and S4†). Dispersion of the distribution also was evaluated through the *s*/*μ* ratio (relative standard deviation) (ESI Fig. S3 and S4†). Low values of *s*/*μ* (in the range of 10^−2^ to 10^−1^) indicate that the average value (*μ*) can be approximated as the actual value of the distribution. Distribution of the distance and angle plots show average distances of 2.70 Å for Cα–H to O atom, 1.83 Å for N1–H to O atom, and 2.23 Å for N2–H to O atom for HAsO_4_^2−^ with DS3 whereas average angular values observed are 137.52° for Cα–H⋯O, 164.37° for N1–H⋯O, and 159.34° for N2–H⋯O. Both the values of distance and angle demonstrate a weak H-bond interaction between the Cα atom and O, while on the other side, H-bonds formed between N1/N2 with O of the ligand are quite strong as the distance observed is <2.5 Å and the X–H⋯O angle is >150°. Both the distance and angle distribution of Cα–H have more variation with respect to those of N1–H and N2–H atoms (ESI Fig. S3 and S4†). This result is in accordance with the H-bond distance and angle as reported in the crystal structures of the ‘C^α^NN’ motif^44^ and that observed for the SO_4_^2−^ and HPO_4_^2−^ ions with the scaffolds.^45,46,48^ Respective average distance values of (Cα)H⋯O, (N1)H⋯O and (N2)H⋯O for the aforesaid ligand interaction of HAsO_3_^2−^ with DS3 are 2.20 Å, 1.99 Å, and 2.07 Å respectively; while the corresponding angles values are 140.60°, 158.95°, and 165.92° in the similar order. This clearly infers better recognition of HAsO_3_^2−^ in comparison to HAsO_4_^2−^ as obtained through binding free energies. Comparing the distribution pattern of interactions between HAsO_3_^2−^ and DS3 with those of HAsO_4_^2−^ and DS3, one would conclude that for HAsO_3_^2−^ distribution of resulting distances and angles of Cα–H and N1–H are attributed to two separate ‘normal-like’ distributions within a range of very small difference (ESI Fig. S3†). Although Ser2-Cα–H of DS3 interacts with the O atom of HAsO_3_^2−^ with an overall average distance of 2.20 Å, upon a keen observation, the entire distribution can be seen as two separate ‘normal-like’ distributions with a distance range of 1.877–1.984 Å and another one of 2.44–2.54 Å (ESI Fig. S3-G†). Similarly, for Lys3-NH, although the overall average distance is 1.9 Å, actually it is a combination of two closely spaced ‘normal-like’ distributions (ESI Fig. S3-H†). However, for HAsO_4_^2−^, a similar interaction produced a single ‘normal-like’ distribution (ESI Fig. S3-A–F†) that is slightly left-skewed. This observation with HAsO_3_^2−^ can be justified from the three-dimensional coordinates of all the generated conformations obtained from the docking algorithm. Although the built-in tool of AutoDock clusters all the 250 poses into a single cluster in terms of binding free energy, a closer look reveals that all the 250 conformations can be forced into two very closely separated clusters depending on the orientations of the ligand. Superposition of the conformations of the two small clusters (being similar in terms of binding free energy) related to HAsO_3_^2−^ differs in RMSD only by ∼0.27 Å. This difference can be understood by the existence of two different states/orientations (S1 and S2) of the ligand (Fig. 1-a1 and a2). The interacting oxygen atoms belonging to these two different conformations of HAsO_3_^2−^ (S1 and S2) are designated as S1O1 and S2O1 (Fig. 1-a1 and a2). Distance (*D*) from the LYS3-N–H and SER2-Cα–H atoms to both S1O1 and S2O1 varies considerably (shown in red and cyan colors, respectively). Hydrogen bonding distance of the ligand oxygen atoms of these two different states/orientations (S1 and S2) of HAsO_3_^2−^ from the main chain C^α^NN participating residues SER2 and LYS3 of DS3 are as follows:

D1: LYS3-N–H to S1O1 (2.1 Å); D2: LYS3-N–H to S2O1 (1.8 Å).

D3: SER2-Cα–H to S1O1 (1.9 Å); D4: SER2-Cα–H to S2O1 (2.5 Å).

A similar pattern of distribution was also observed for the angle ∠X–H⋯O constituted by Ser2 and Lys3 with HAsO_3_^2−^ (ESI Fig. S3-J and K†). Such an interesting observation of having two conformations (S1 and S2) for HAsO_3_^2−^ (Fig. 1-a1 and a2) but not for HAsO_4_^2−^ can perhaps be explained by the presence of the lone pair of electrons on As in HAsO_3_^2−^ (oxidation state of As +3) which allows flipping of the pseudotetrahedral geometry of HAsO_3_^2−^ generating two different, but energetically closely related, conformations. Obviously, such structural tumbling/flipping is not feasible for HAsO_4_^2−^ due to its rigid tetrahedral geometry. Further, in the case of DS1, due to presence of flexible Gly in the second position, such an anion dependent distinct feature may not be clearly observed (ESI Fig. S4†). Glycine, having no side chain, can use both its enantiotropic hydrogens (connected at the Cα-atom) to recognize the O-atom of the ligand through hydrogen bonding. Inclusion of flexibility in the anion recognition scaffold of the peptide gives less variation in the bonding parameters although producing a slight translation in the distribution range (either left or right skewed) for both anions. This observation leads to a conclusion that orientation of the ligand (anion) is not alone responsible for the perturbation in the interaction pattern; the sequence/3D-structure of the host peptide participating in the recognition would also affect the interaction parameters which are reflected in the binding free energy. It is also noted that, apart from the main chain hydrogen bond, there is also an add-on contribution from the side chain of the Ser residue(s) of DS3 in ligand binding (confirmed in the Molecular dynamics simulation section) which increases stability of the interaction.

In a nutshell, from the docking analysis one can conclude that effectiveness of the recognition of arsenic anions depends on local information embedded in the peptide sequence and conformation as observed for the sulfate and the phosphate ions. Further, the results obtained distinctly identify that in a context free system the designed peptide scaffolds (DS1 and DS3) recognize As ligands favorably over the naturally occurring ‘C^α^NN’ sequences. These designed scaffolds, DS1 and DS3, are considered as potentially active to act as scavengers of As for its removal. However, as in aqueous solution, HAsO_3_^2−^ exists in a much higher than physiological pH (p*K*_2_ of H_3_AsO_3_ is 12.13), therefore, further experiments were focused on HAsO_4_^2−^ (existing at physiological pH) only.

### 3.4 Mass spectrometry

**Fig. 2:**
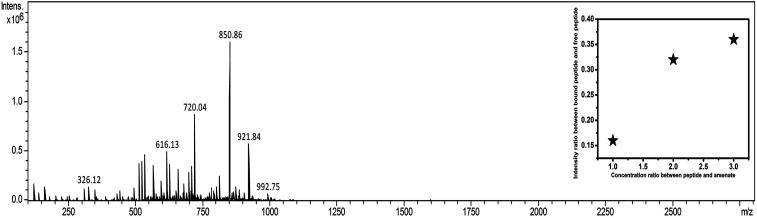
ESI-MS data of arsenate (HAsO_4_^2−^) added to peptide (DS3). Binding of arsenate with the peptide DS3 was established through appearance of the *m*/*z* value of 921.9. Moreover, un-reacted peptide can be identified by the *m*/*z* value of 850.9 (inset shows the scatter plot of intensity ratio between arsenate-bound peptide and free peptide as a function of added arsenate ion concentration with respect to the peptide. As the concentration of added arsenate was increased, the ratio was enhanced from 0.15 to 0.35 and subsequently becomes saturated when arsenate is added at 3 times than that of the peptide).

Appearance of *m*/*z* values at 1705.1, 1727.1, and 1743.1 corresponding to M + H^+^, M + Na^+^, and M + K^+^ added species, respectively, in the positive mode of MALDI validated the existence of DS3 (MW 1704). Further, appearance of no other *m*/*z* values with appreciable intensity clearly justified the purity of the sample. Molecular association of arsenate with peptide DS3 (added as Na_2_HAsO_4_ in 1 : 3 M ratio of peptide : arsenate) was established in the negative mode of ESI-MS [H_2_O/CH_3_CN (1 : 1) + 0.1% NH_3_]. Appearance of a peak corresponding to *m*/*z* value 921.9 established the binding of arsenate (HAsO_4_^2−^) with the peptide DS3 (Fig. 2). Moreover, isotopic distribution of peaks corresponding to differences in *m*/*z* values of 0.5 (921.4, 921.9, and 922.4; data not shown) signified recognition of the divalent arsenate anion (HAsO_4_^2−^) by the peptide DS3 and validated the observation of the molecular docking experiment. However, the *m*/*z* value at 850.9 corresponds to un-reacted DS3.

### 3.5 Circular dichroism spectroscopy

**Fig. 3:**
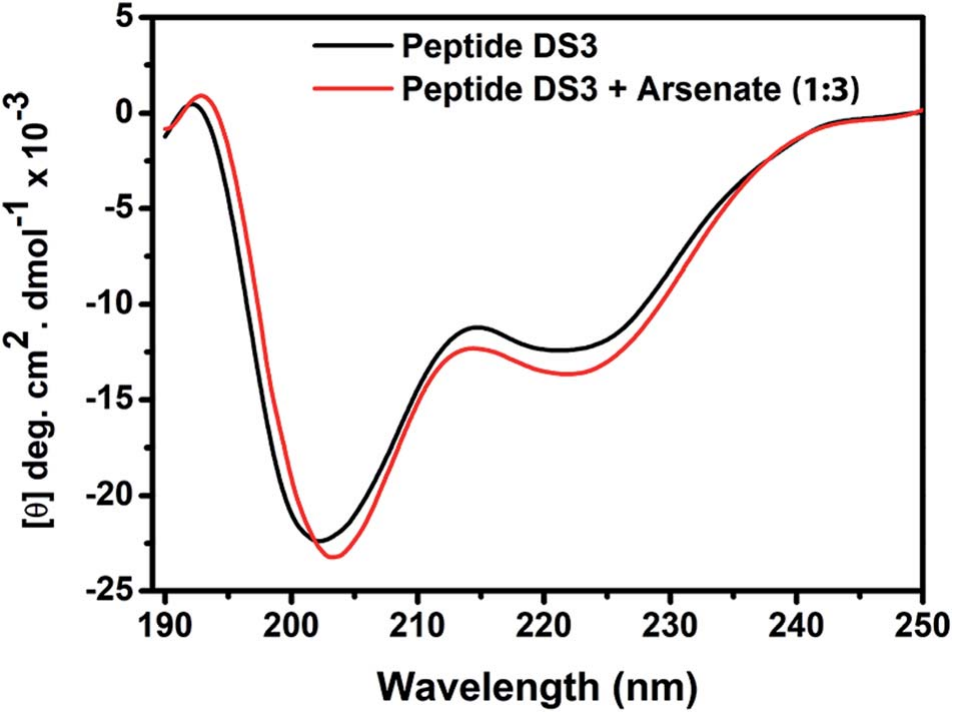
CD spectrum of the peptide DS3 (represented as black line) along with its arsenate (HAsO_4_^2−^) added (1 : 3 peptide : arsenate) species (represented as red line) measured at 298 K, showing enhancement of CD signals for both n–π* (∼222 nm) and 
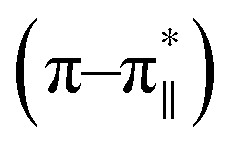
 (∼208 nm) transitions after addition of arsenate to peptide DS3.

In order to explore conformational changes of the peptide DS3 upon interaction with arsenate, if any, CD spectra of the peptide were recorded in absence as well as in presence of added arsenate ions at room temperature (25 °C). Peptide DS3 in a fully aqueous condition showed characteristic double minima at ∼202 
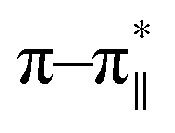
 and 222 (n–π*) nm (Fig. 3) corresponding to a helical signature of the peptide,^75^ which may arise from the contributions of admixture of 3_10_- and α-helices. After addition of arsenate ions (added as Na_2_HAsO_4_ in 1 : 3 M ratio of peptide : anion), a CD spectrum of the peptide also was recorded. The CD spectrum of the arsenate-added peptide (Fig. 3) showed enhanced CD signals in both the 
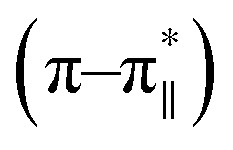
 and n–π* transitions (especially n–π* transition at 222 nm). This signified an increment of helical population of the peptide DS3 upon interaction with the arsenate ions as observed for the SO_4_^2−^ and the HPO_4_^2−^ ions with the naturally occurring ‘C^α^NN’ motif sequences.^45,47^ In order to quantify the increment in helicity upon interaction with the arsenate ion, the ratio *R*_2_
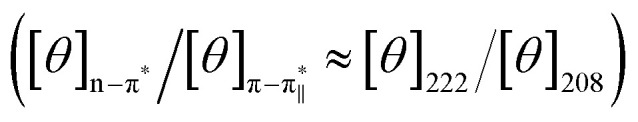
^76–78^ was calculated. Upon addition of HAsO_4_^2−^ to DS3, an enhanced magnitude of *R*_2_ (from 0.50 to 0.62) of DS3 along with a ∼2 nm bathochromic shift at 
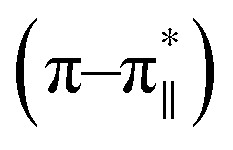
 transition (∼204 nm → ∼206 nm) strongly recognized the interaction of the anion with the peptide along with the enhancement of helicity upon recognition and corroborated the output of the molecular docking experiment.

### 3.6 Isothermal titration calorimetric (ITC)

**Fig. 4:**
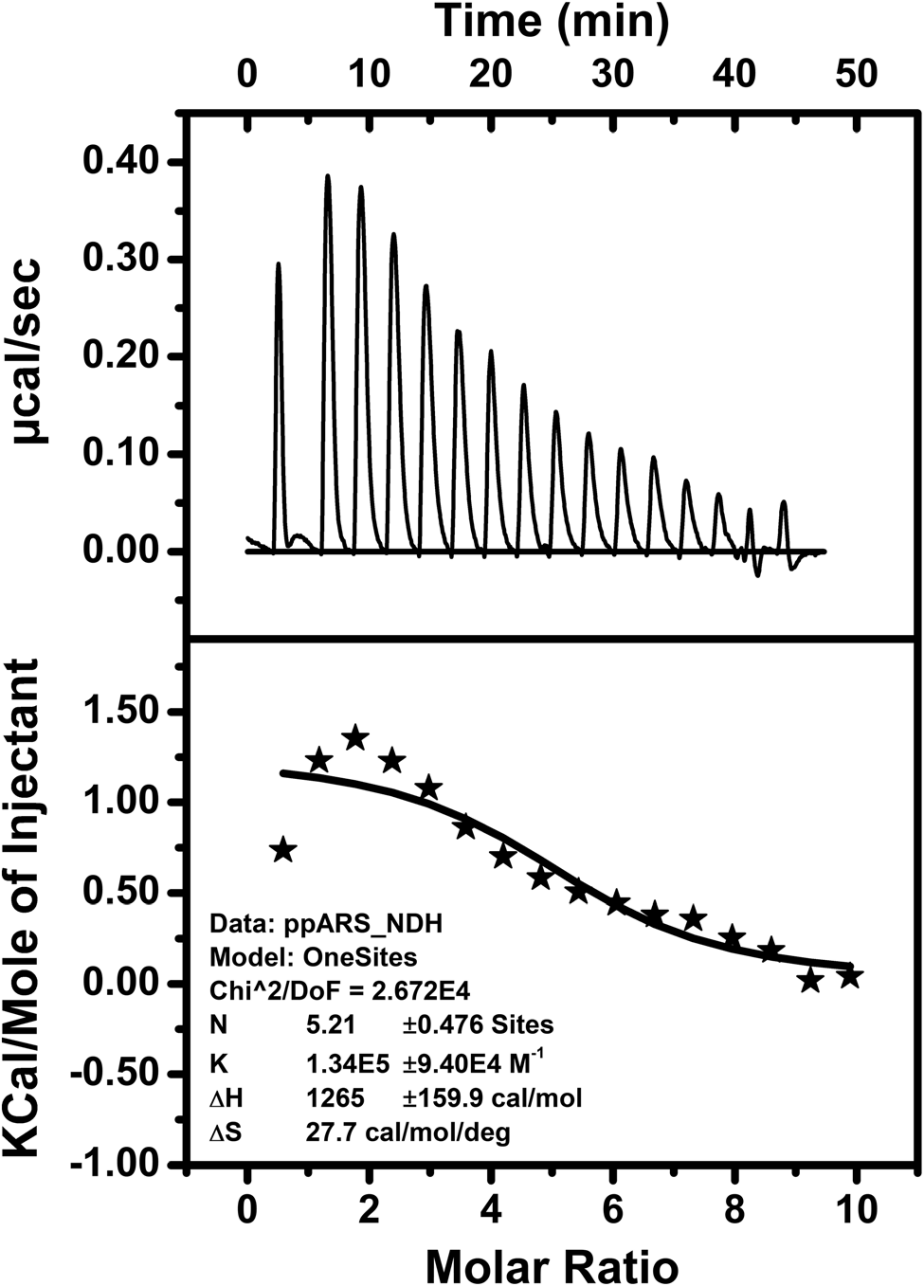
ITC figure representing the peptide (DS3)–arsenate interaction measured using Microcal ITC200 (Malvern) at 25 °C. Top panel represents the raw thermogram of the interaction while the lower panel represents the best fit to a single site model along with the thermodynamic parameters.

The thermodynamic parameters of bio-molecular interactions can be described using isothermal titration calorimetry (ITC).^79^ These interactions involve a wide range of molecular associations, such as metal ions binding to proteins,^80–82^ protein–protein interactions,^83^ or ligand–protein complex formations.^84,85^ Here, interaction of the peptide DS3 with arsenate (HAsO_4_^2−^) was further studied through ITC. The ITC data of binding interactions between peptide DS3 and arsenate, shown in Fig. 4, established an endothermic mode of interaction. From the raw ITC thermogram of peptide DS3 titrated against arsenate (Fig. 4), the associated thermal changes were plotted against the molar ratio. To substantiate binding affinity of the peptide for arsenate, the dissociation constant (*K*_d_) was measured from the plotted curve. The observed *K*_d_ value of ∼8 μM signified an appreciable amount of interaction of arsenate with the peptide DS3, which is even better than the interaction involving peptide CPS224Ac/CPS226 (containing naturally occurring ‘C^α^NN’ motif) with sulfate.^47^ From the magnitude of the thermodynamic parameters like enthalpy (Δ*H*: 1265 cal mol^−1^) and entropy (Δ*S*: 27.7 cal mol^−1^ deg^−1^), the free energy (Δ*G*) of the interaction was calculated. The observed physical parameters dictate that the interaction is an entropy driven process (positive Δ*S* value) having free energy (Δ*G*) of −7 kcal mol^−1^. This amount of free energy of the system corroborates an appreciable and spontaneous interaction between the peptide DS3 and arsenate.

### 3.7 Molecular dynamics experiment

**Fig. 5:**
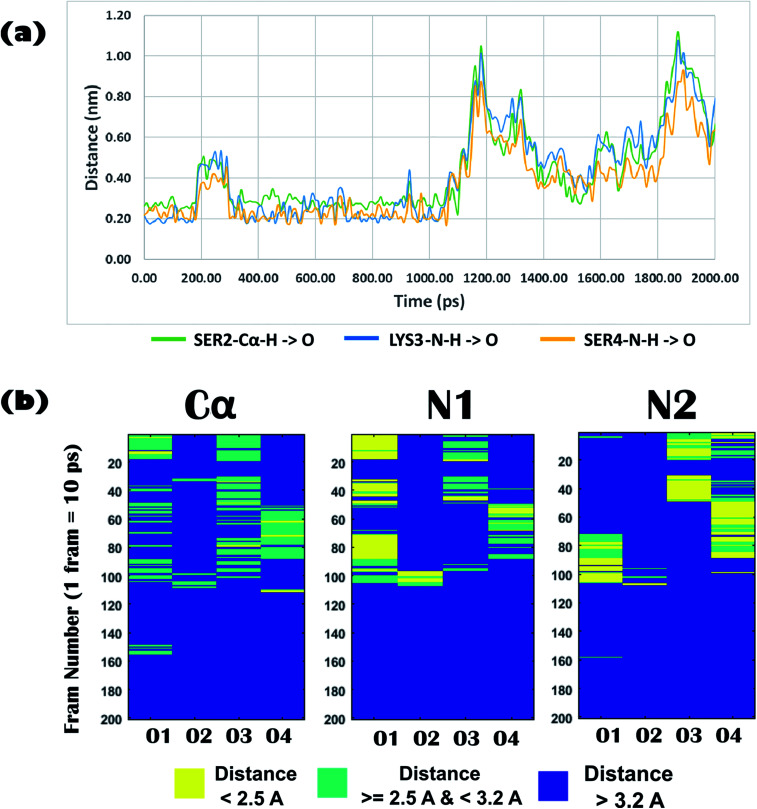
(a) Time dependent variation of distance of the ‘C^α^NN’ motif residues (Cα of Ser2, main-chain N–H of Lys3, main-chain N–H of Ser4) of DS3 and oxygen atoms of Arsenate ligand (HAsO_4_^2−^). Up to 2 ns of the MD trajectory is shown in this figure where it can clearly be seen that the ion HAsO_4_^2−^ interacts with the C^α^NN region of DS3 up to 1.06 ns. (b) Distance profile of O atoms (up to 2 ns) of ligand (HAsO_4_^2−^) from the Ser2-Cα–H, Lys3-N1–H, and Ser4-N2–H of ‘C^α^NN’ motif residues of DS3 in time dependent manner resembling the C^α^NN-HPO_4_^2−^ interaction as reported in literature [*X* axis: oxygen atom number of the ligand HAsO_4_^2−^; *Y* axis: frame number (1 frame = 10 ps, *i.e.*, up to 2000 ps to 2 ns is shown)].

**Fig. 6:**
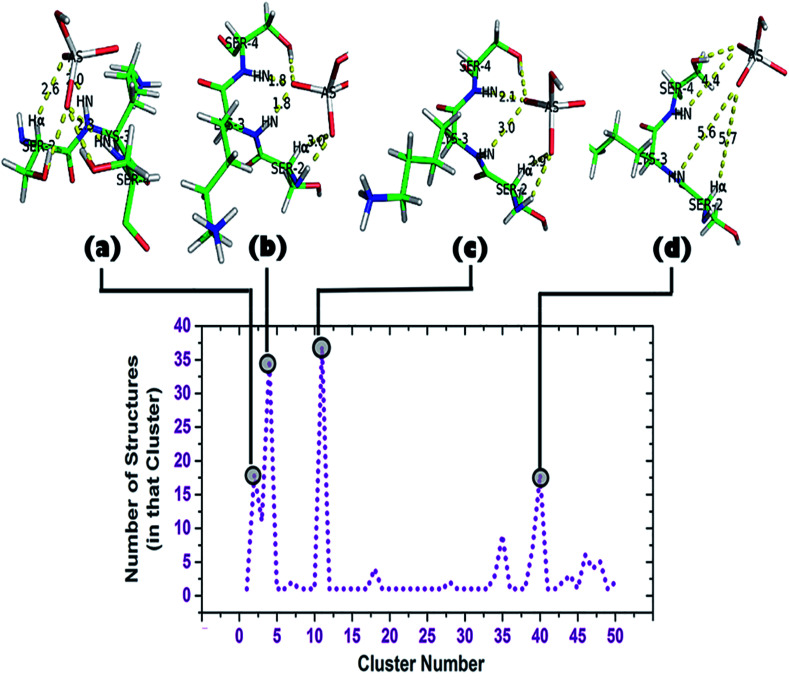
Cluster analysis of the trajectory up to 2 ns (DS3-HAsO_4_^2−^). Total of 50 clusters were found, each having a ‘representative structure’ and number of structures belonging to that ‘representative structure’ are shown beside. Top clusters (clusters containing largest numbers of structures: 2, 4, 11, and 40) and their corresponding conformations are shown.

Biophysical experiments (using mass spectrometry, CD spectroscopy, and ITC) clearly indicated the recognition of arsenate by the DS3 peptide. This interaction information of the peptide DS3 and arsenate at the atomic level can further be retrieved as a function of time from the Molecular Dynamics (MD) simulation experiment, as the previous experiments gave only time averaged results. Investigation of interactions between the arsenate (HAsO_4_^2−^) ions and DS3 portray a major similarity in the signature of the interactions of the naturally occurring ‘C^α^NN’ motif and SO_4_^2−^ ions^46^ along with DS3 and SO_4_^2−^ ions.^48^ This may be due to the fact that since both the anions have similar stereochemistry along with net charge content, the inner line 3d orbital of As makes its van der Walls radius larger than that of either phosphorous or sulfur. Previous investigations using the simulation experiment of DS3 with sulfate suggested a more stable binding of the sulfate ion with DS3 (∼4.9 ns residence time of SO_4_^2−^)^48^ than with the natural ‘C^α^NN’ sequence present in the CPS224Ac peptide (residence time of SO_4_^2−^ ion only 400 ps).^46^ Here, the time dependent behavior of the HAsO_4_^2−^ ion with the Ser2-Lys3-Ser4 sequence of the DS3 scaffold revealed that the anion interacts with the core of the ‘C^α^NN’ motif up to ∼1.06 ns (1 ns and 60 ps) mediated through a hydrogen bond (Fig. 5a). Time dependent behavior of the torsion angles and other related parameters of interactions of arsenate (HAsO_4_^2−^) with the scaffold (DS3) is briefed in the underlying study. Average hydrogen bonding (H-bond) distance between the O-atoms of the anion and Ser2-Cα–H is 3.0 Å (±0.6), whereas that with Lys3-N1–H and Ser4-N2–H are 2.6 Å (±0.9) and 2.4 Å (±0.6), respectively (up to the residence time of ligand with ‘C^α^NN’, *i.e.*, 1.06 ns) (Fig. 5b). Distance of the Cα atom with the ligand was more, as expected, than that of N1 and N2, indicating the comparatively weaker nature of this interaction. This is at par with the observation obtained from the molecular docking experiments. However, the standard deviation for the N1 was the largest. Along with the interactions between the main chain atoms of the ‘C^α^NN’ motif of the peptide DS3 with HAsO_4_^2−^, interactions from the side chain were also observed as follows: Ser4-OG (side-chain oxygen) (Fig. 6a) 1350–1450 ps, 1590–1780 ps, 2050–2210 ps; LYS3-N–H (side-chain –NH): 2380–3780 ps. Terminal presence of the Ser2 facilitated attainment of various backbone torsions relatively more freely maintaining the hydrogen bond (facilitated *via* the side-chain HG1 atom) with the ligand. This makes the N1 to point obliquely which may be one of the reasons for its greater distance fluctuation with the anion. This was not observed with the molecular docking because, due to rigid docking, the geometry of the host molecule (peptide) is fixed. Distance and torsion parameters for the Ser4 residue were mostly constant in the simulated trajectory because it has less freedom when present at the cap of the N-term of helical ABGY. An unusual behavior of torsion fluctuation was noticed in the Ser2 residue where it switches to a left handed α-helical state (αL) from 200 ps to 4200 ps and again from approximately ∼15 310 ps to 17 110 ps, otherwise, it remained in the β conformation (ESI Fig. S5†). However, effects of arsenate on this transition are not clearly understood as in the case of torsion transition for a later time interval, where the ligand was distantly located (∼60 Å) from the scaffold. In contrast to the previous study, the Lys3 residue remained mostly in an alpha helical (right-handed) state throughout the entire simulation time (ESI Fig. S5†) which may assist with recognizing the anion as the N1 residue in the ‘C^α^NN’, which appears with α-helical geometry upon anion recognition. Within this helical adapted state, participation of the main-chain N–H atom of Lys3 and Ser4 in binding the ligand is noticeable (Fig. 6). Considering the first 5 ns of simulation trajectory, it can be seen that Ser2 mostly occupies the αL torsion state (as mentioned earlier). Up to 5 ns the average torsion angle values for the motif in DS3 are as follows: Ser2: phi (*ϕ*) is 41.12 (±52.29), psi (*ψ*) is 64.32 (±38.36), Lys3: phi (*ϕ*) is −71.06 (±17.19), psi (*ψ*) is −35.86 (±12.44), Ser4: phi (*ϕ*) is −66.58 (±7.87), and psi (*ψ*) is −39.56 (±7.85) (ESI Fig. S5†). Fluctuation of the terminal amino acid residues in the protein backbone are obvious. But, with up to ∼1.06 ns the torsion angles of Ser2 remain very stable having phi (*ϕ*) of 67.11 (±14.19) and psi (*ψ*) of 60.42 (±27.14) and maintaining αL geometry (ESI Fig. S5,† inset) indicating constraints for association of anions. Alongside, torsion angular values for Lys3 and Ser4 up to 1.06 ns are as follows: Lys3, phi (*ϕ*) is −90.87 (±23.62) and psi (*ψ*) is −22.95 (±14.53); Ser4 – phi (*ϕ*) is −65.86 (±6.88), and psi (*ψ*) is −40.77 (±6.44).

A cluster analysis was performed on the truncated simulated trajectory (up to 2 ns) by the default ‘*gmx cluster*’ tool available within GROMACS 5.1.2 to identify the principal ligand bound with the motif under investigation. Each cluster represents a particular conformational state and also shows the number of structures belonging to that state. It helps to decide the top clusters with most favorable geometry comprising a larger number of participant states. Participation of Ser2 can be seen in the top two representative structures of the top two clusters (Fig. 6b and c). A total of 50 clusters were considered from the cluster analysis with a RMSD range of 0.06374–0.36899 Å and representative structures from the most populated clusters are shown in Fig. 6. Cluster 2, having 18 structures, shows a typical anion binding pattern with the Ser2-Lys3-Ser4 residues where one oxygen interacts with Cα–H and N1–H, while N2–H interacts with another oxygen of arsenate (HAsO_4_^2−^) resembling the naturally occurring ‘C^α^NN’ motif with phosphate.^44^ A distant location of a ligand from the scaffold can be seen in cluster 40 which captures the ejection of the arsenate. Another observation is the participation of a side chain of lysine residues in the simulation trajectory, contrary to the DS3-sulfate simulation^48^ where, after ejection of the anion, the anion remains free in the system. Within time steps 32 600–41 000 ps (approx.) arsenate was found to interact with both the Lys8 and Lys11 side-chains. Apart from the 1.06 ns interval of HAsO_4_^2−^–DS3 interaction, the Lys3 side-chain again is very near to the ligand within a 45 000–49 000 ps (approx.) interval. A distance profile of the HAsO_4_^2−^ ligand from all the residues of the DS3 peptide is also shown (ESI Fig. S6†) to decipher the aforementioned feature of Lys residues. As mentioned previously, the bulky nature of arsenic (larger atomic radius) in comparison to sulfur may enhance the influences of a charged side-chain of lysine on the ligand in the overall conformational landscape by decreasing the mobility of HAsO_4_^2−^. Though SO_4_^2−^ has a higher affinity towards the ‘C^α^NN’ segment of DS3, in a competitive binding environment the DS3-HAsO_4_^2−^ interaction will be favored because of retention ability by the ‘C^α^NN’ segment as well as the Ser and Lys side-chains. This motivated us to conclude that DS3 can act as a scavenger of HAsO_4_^2−^.

## 4 Conclusions

The environmental pervasiveness of arsenic is a matter of deep concern^13^ due to its high occurrence in groundwater as arsenate or arsenite (along with its methylated derivatives), which interfere with metabolic pathways and normal cellular activities. Site specific ion recognition is the major driving force behind various biological processes^86–89^ where loop regions of proteins with higher solvent accessible surface areas^90^ interact with As ions utilizing various motifs. Peptide-based model scaffolds mimicking the natural protein-ligand interaction provide rich information regarding binding affinity, mode, and the contribution of motif geometry. Utilizing this concept, we initiated an effort to study peptide-arsenate/arsenite interactions that ultimately could be used for scavenging arsenic anions. In the present study, the participation of main-chain Cα_*i*−1_–H, N_*i*_–H, N_*i*+1_–H residues of consecutive amino-acids, based on the ‘C^α^NN’ motif was utilized to recognize different anions of arsenic. Comparable stereochemistry between HAsO_4_^2−^ and HPO_4_^2−^ acted as the motivating spirit and both of the anions showed similar influence on the designed ‘C^α^NN’ motif in a context-free system that had similar interacting properties with the conserved binding pattern. Further, a kinetic model of interaction was validated through a molecular dynamics simulation of the DS3-HAsO_4_^2−^ docked conformation (having well-inferred the binding ability of DS3 in comparison to other peptides) where the residence time for HAsO_4_^2−^ was 1.06 ns for the scaffold DS3, which is quite appreciable. Though no interaction with a side chain was found for sulfate with DS3 once the anion left the ‘C^α^NN’ (Ser2-Lys3-Ser4) segment, some extent of interaction was found for HAsO_4_^2−^ through the participation of side-chains on polar residues that enhance the interactions between HAsO_4_^2−^ ions and the peptide scaffold (DS3). Reduced flexibility of movement for the As ligand, due to its larger size and diffused electron density, provided an additional effect toward the retention of the anions. All these data strongly suggest that the ‘C^α^NN’-based scaffold DS3 can well recognize anions of arsenic and be their scavenger through attachment in a water filtration unit. Ligand binding abilities by the conserved geometry presented in this work, with the help of a ‘natural replica’ of the ‘C^α^NN’ motif, contribute significantly towards understanding the context of free peptide–ligand recognition, which may be utilized for scavenging toxic anions through peptidomimetics.

## Conflicts of interest

There are no conflict to declare.

## Supplementary Material

RA-009-C8RA08225A-s001
