# Supplementary material for: Interaction landscape of a ‘CαNN’ motif with arsenate and arsenite: a potential peptide-based scavenger of arsenic
Source: RSC Adv. 2019 Jan 9;9(2):1062–74. doi: 10.1039/c8ra08225a (PMC9059529; doi:10.1039/c8ra08225a)
Supplement: RA-009-C8RA08225A-s001 [file RA-009-C8RA08225A-s001.pdf]

Electronic Supplementary Material (ESI) for RSC Advances.
This journal is © The Royal Society of Chemistry 2018

Supporting Information

Interaction landscape of ‘C^{α}NN’ motif with Arsenate and Arsenite: towards identifying peptide based scavenger of Arsenic

Subhankar Sahu, Tridip Sheet and Raja Banerjee*
Maulana Abul Kalam Azad University of Technology
(Formerly Known as West Bengal University of Technology),
Salt Lake, Kolkata 700064, West Bengal, India

Correspondence

Raja Banerjee, Professor Department of Biotechnology and Head Department of Bioinformatics, Maulana Abul Kalam Azad University of Technology, West Bengal, (Formerly known as West Bengal University of Technology) BF-142, Sector-1, Salt Lake, Kolkata- 700064, India.
Email: ban_raja@yahoo.com; banraja10@gmail.com

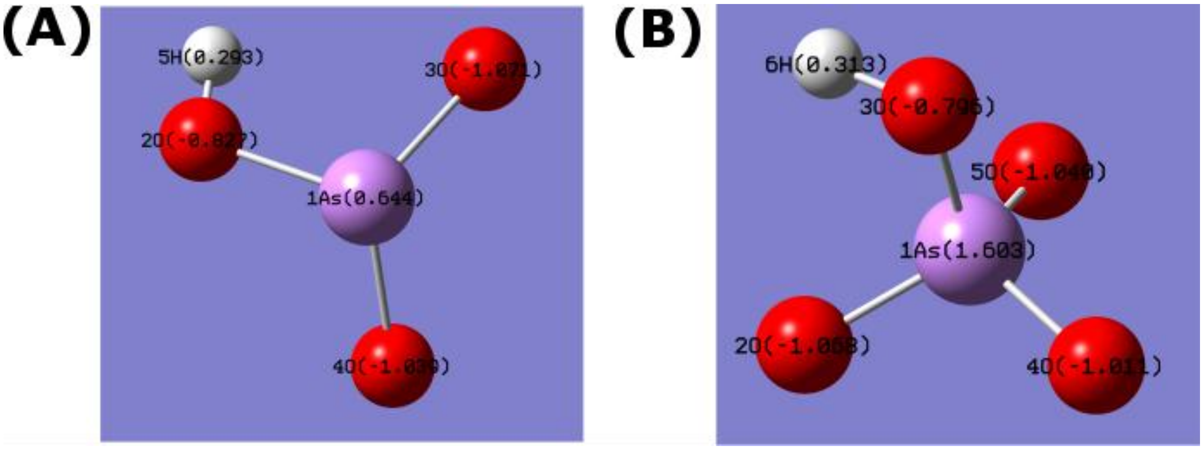

Supplementary Figure S1. Partial charge distribution of (A) $HAsO_3^{2-}$ (B) $HAsO_4^{2-}$. (Partial charge is calculated by semi-empirical PM6 Hamiltonian basis set by Gaussian 09 Suit.)

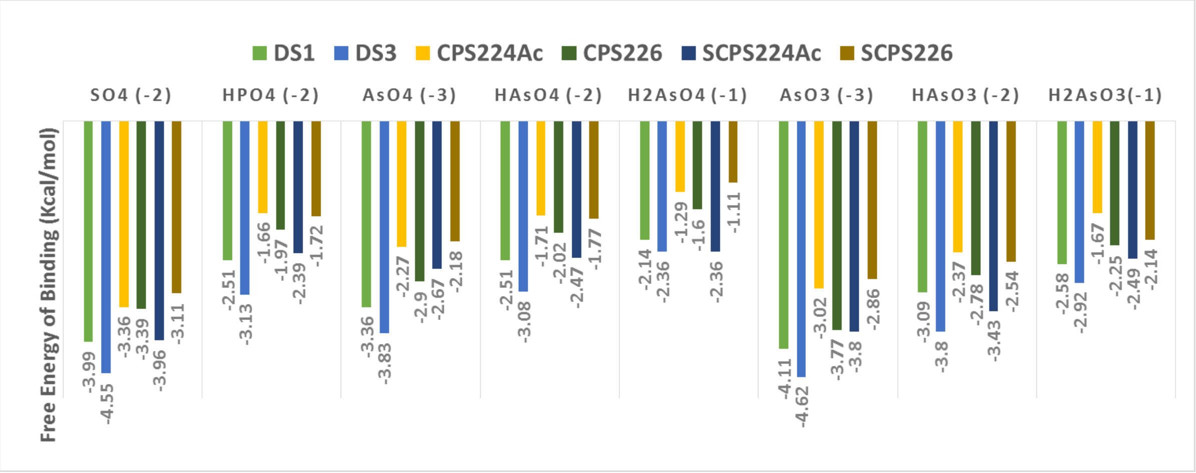

Supplementary Figure S2. Bar plot displaying the comparative free energy of binding of all the ligands and chimeric peptides used in the study from docking analysis. Peptides and different ligand combinations are designated with different color bar.

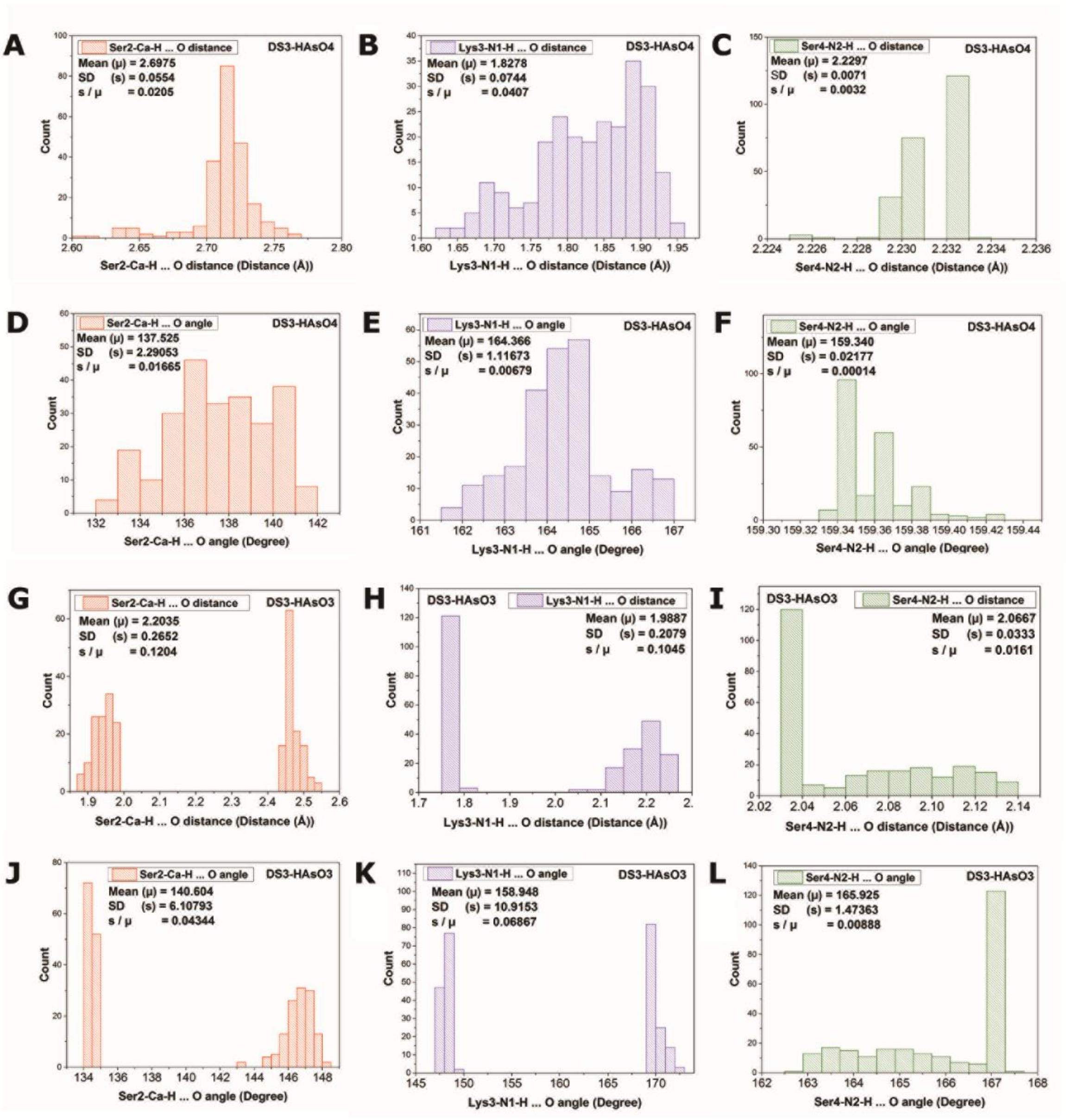

Supplementary Figure S3. Histogram plot of Hydrogen bond Distance & Angle distribution of DS3 with $HAsO_4^{2-}$ (**A-F** , upper half of the image) and $HAsO_3^{2-}$ (**G-L** , lower half of the image) as ligand from the Molecular docking. Mean (μ), standard deviation (s), and s/μ values are given on each plot.

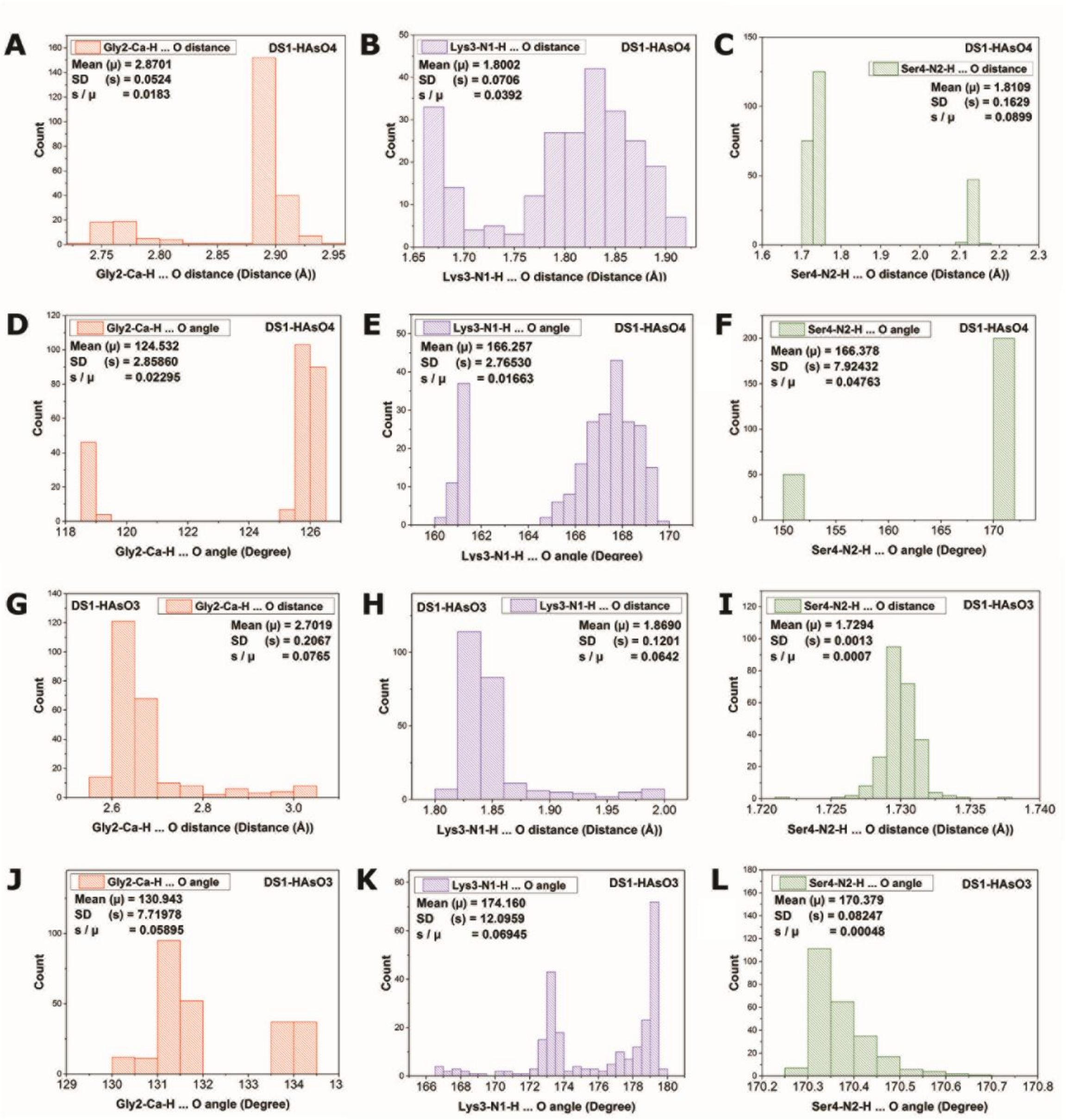

Supplementary Figure S4. Histogram plot of Hydrogen bond Distance & Angle distribution of DS1 with $HAsO_4^{2-}$ (**A-F** , upper half of the image) and $HAsO_3^{2-}$ (**G-L**, lower half of the image) as ligand from the Molecular docking. Mean (μ), standard deviation (s), and s/μ values are given on each plot.

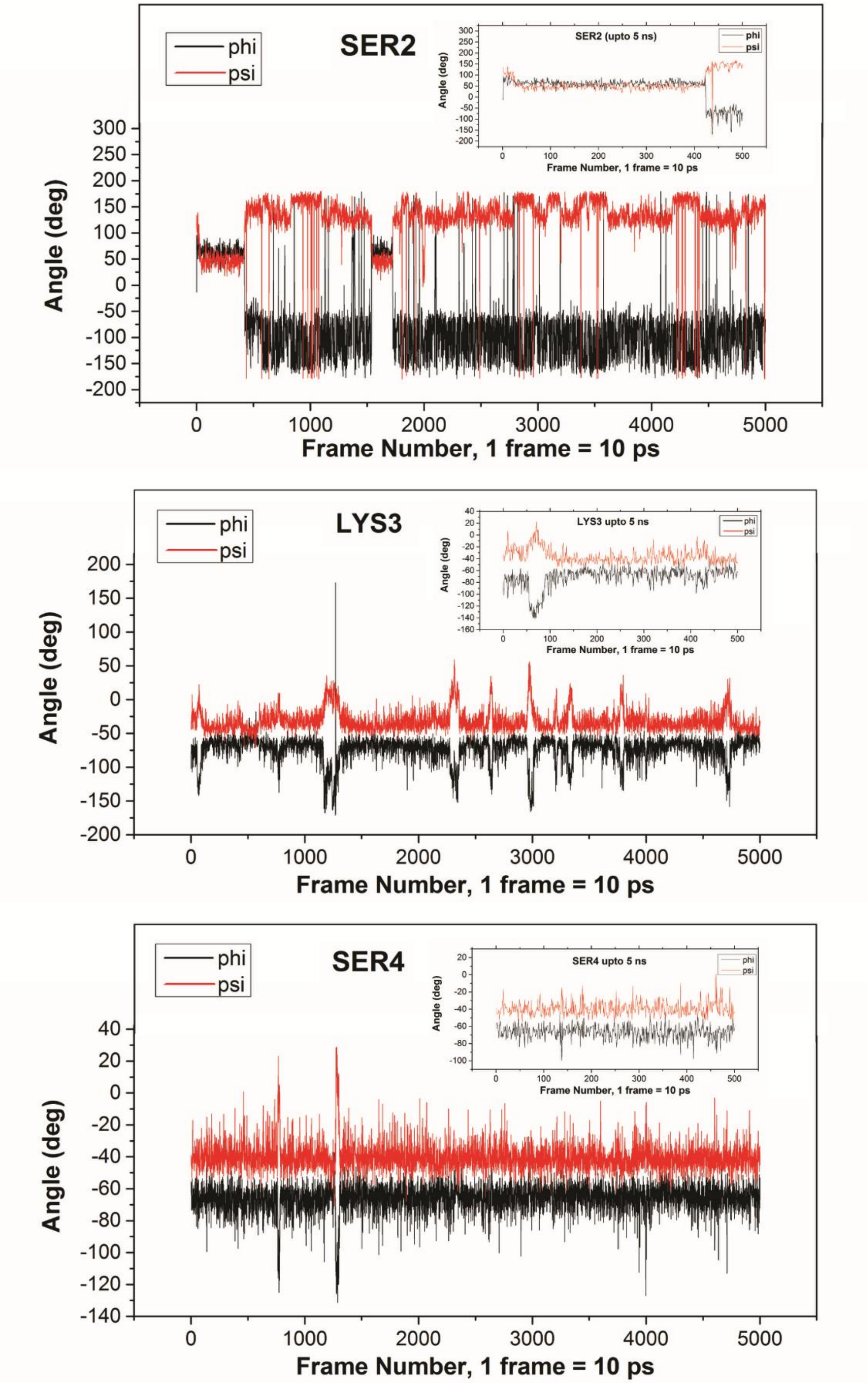

Supplementary Figure S5. Time dependent variation of Torsion angles (for entire 50 ns simulation, DS3 & $HAsO_4^{2-}$ as ligand) of 'CαNN' motif residues of DS3 peptide. X axis: Frame number, 1 frame = 10 ps. Y axis: Angle (degree). Up to 5 ns of the simulation trajectory is shown as inset.

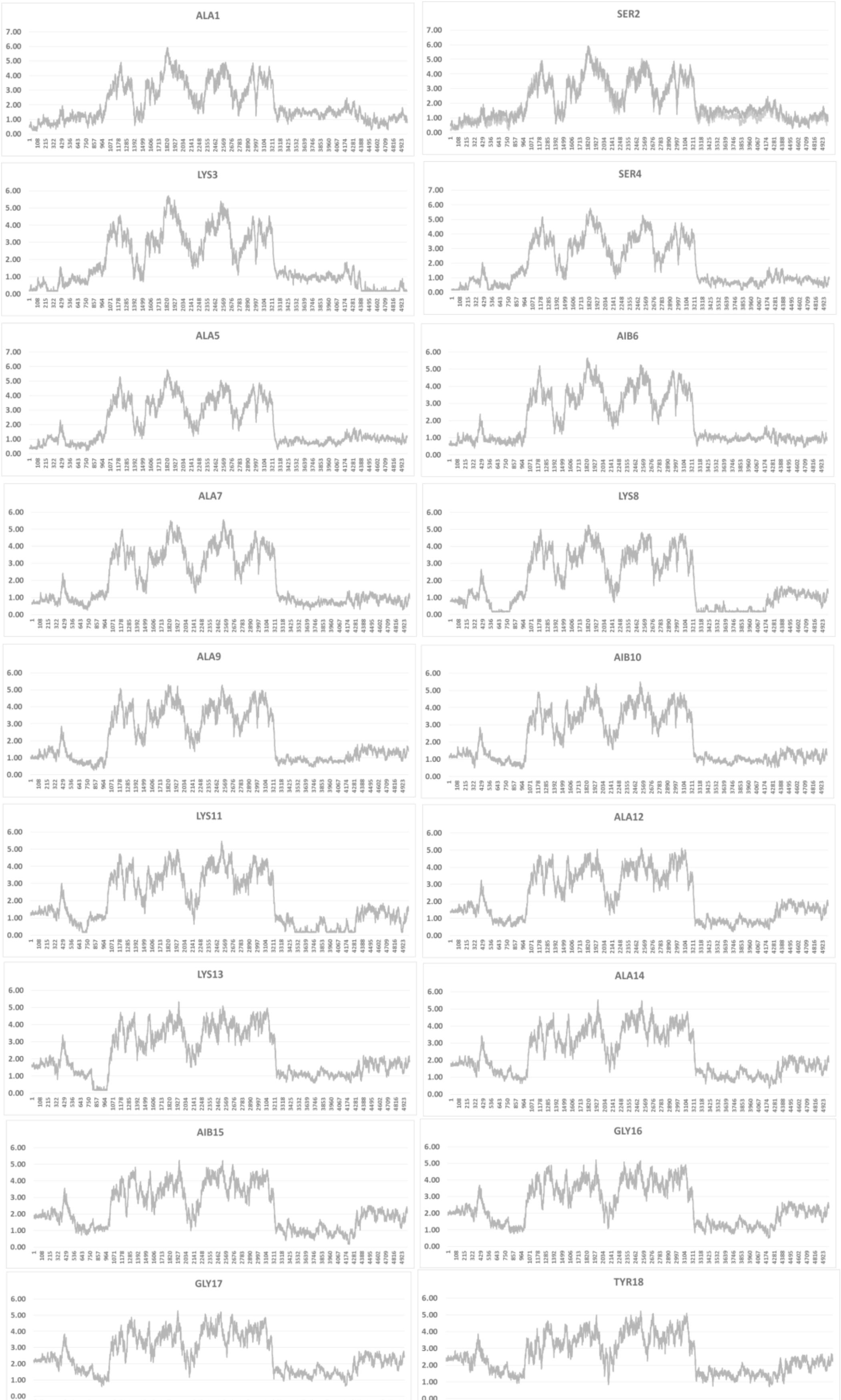

Supplementary Figure S6 Distance between all the residues of DS3 and $HAsO_4^{2-}$ ion. Corresponding residue names are written on the plot. X-axis : Frame number (1 frame = 10 ps). Y-axis : Distance (nm).
